# The *stb* Operon Balances the Requirements for Vegetative Stability and Conjugative Transfer of Plasmid R388

**DOI:** 10.1371/journal.pgen.1002073

**Published:** 2011-05-19

**Authors:** Catherine Guynet, Ana Cuevas, Gabriel Moncalián, Fernando de la Cruz

**Affiliations:** IBBTEC, Instituto de Biomedicina y Biotecnologia de Cantabria (CSIC-UC-SODERCAN), Facultad de Medicina, Universidad de Cantabria, Santander, Spain; Universidad de Sevilla, Spain

## Abstract

The conjugative plasmid R388 and a number of other plasmids carry an operon, *stbABC*, adjacent to the origin of conjugative transfer. We investigated the role of the *stbA*, *stbB*, and *stbC* genes. Deletion of *stbA* affected both conjugation and stability. It led to a 50-fold increase in R388 transfer frequency, as well as to high plasmid loss. In contrast, deletion of *stbB* abolished conjugation but provoked no change in plasmid stability. Deletion of *stbC* showed no effect, neither in conjugation nor in stability. Deletion of the entire *stb* operon had no effect on conjugation, which remained as in the wild-type plasmid, but led to a plasmid loss phenotype similar to that of the R388Δ*stbA* mutant. We concluded that StbA is required for plasmid stability and that StbA and StbB control conjugation. We next observed the intracellular positioning of R388 DNA molecules and showed that they localize as discrete foci evenly distributed in live *Escherichia coli* cells. Plasmid instability of the R388ΔΔ*stbA* mutant correlated with aberrant localization of the plasmid DNA molecules as clusters, either at one cell pole, at both poles, or at the cell center. In contrast, plasmid molecules in the R388ΔΔ*stbB* mutant were mostly excluded from the cell poles. Thus, results indicate that defects in both plasmid maintenance and transfer are a consequence of variations in the intracellular positioning of plasmid DNA. We propose that StbA and StbB constitute an atypical plasmid stabilization system that reconciles two modes of plasmid R388 physiology: a maintenance mode (replication and segregation) and a propagation mode (conjugation). The consequences of this novel concept in plasmid physiology will be discussed.

## Author Summary

The ability of bacteria to evolve and adapt to new environments most often results from the acquisition of new genes by horizontal transfer. Plasmids have a preponderant role in gene exchanges through their ability to transfer DNA by conjugation, a process that transports DNA between bacteria. Besides, plasmids are autonomous DNA molecules that are faithfully transmitted to cell progeny during vegetative cell multiplication. In this study, we report a system composed of two proteins, StbA and StbB, which act to balance plasmid R388 physiology between two modes: a maintenance mode (vertical transmission) and a propagation mode (horizontal transmission). We demonstrate that StbA is essential to ensure faithful assortment of plasmid copies to daughter cells. In turn, StbB is required for plasmid R388 adequate localization for conjugation. This is the first report of a system which reconciles plasmid segregation and conjugation. Furthermore, R388 belongs to the IncW family of conjugative plasmids, which are of particular interest due to their exceptionally broad host range. We show that the StbAB system is conserved among a wide variety of conjugative plasmids, mainly broad host range plasmids. Thus, the Stb system could constitute an interesting therapeutic target to prevent the spread of adaptive genes.

## Introduction

Transmissible plasmids contribute greatly to the plasticity of bacterial genomes and to the acquisition of genetic traits by host cells through the collective carriage of adaptive genes, including antibiotic resistance and virulence genes, and through the ability to disseminate them by conjugation [1]. Horizontal gene transfer may thus increase the adaptability of bacteria to changing environmental conditions, which is dramatically exemplified by the emergence and spread of multiple antibiotic-resistance plasmids in and between potentially pathogenic bacteria. Conjugative plasmids are transmitted both vertically to daughter cells and horizontally to other strains or species. Vertical transmission requires timely controlled replication and faithful assortment (segregation) of sister plasmid copies to daughter cells. Segregation occurs by a range of different mechanisms including control of copy number, resolution of multimeric plasmid molecules and, in the case of most low copy number plasmids, active segregation (partition). Partition (Par) systems ensure efficient distribution of plasmid molecules to each daughter cell during division (for reviews: [2]–[5]). They are composed of a *cis*-acting centromere-like site and two proteins, a nucleotide-binding cytomotive protein and a centromere-binding adaptor protein. Stable inheritance requires that these proteins form a partition complex on the centromere. The *par* centromere locus and the Par proteins are encoded by sets of homologous genes in various plasmids, phages, and chromosomes.

The mechanism of plasmid conjugation in gram negative bacteria has been well characterized (for a review: [6]). The overall process is accomplished by two functional multiprotein complexes encoded by two gene clusters: the set of mobility genes (MOB), involved in conjugative DNA processing, and the mating pair formation cluster (MPF), encoding the nucleoprotein transport apparatus. These two systems are connected by the coupling protein (T4CP). The MPF is a type IV protein secretion system and implies the assembly of a multiprotein complex at the bacterial membrane [7]. Conjugal DNA processing involves the formation of a nucleoprotein complex called relaxosome at the cognate origin of conjugative transfer (*oriT*). The relaxase catalyses a DNA strand- and site- specific cleavage at the *nic* site of *oriT*, and remains covalently attached to the 5′ end of the cleaved single-stranded (ss) DNA [6]. Complementary-strand synthesis is initiated from the free 3′ end of the cleaved strand through rolling-circle replication. The relaxase-ssDNA complex interacts with the T4CP, which guides the transferred strand through the DNA transfer apparatus formed by the MPF proteins throughout the membrane and into the recipient cell [8], [9]. Fundamental questions remain unanswered concerning the spatiotemporal coordination of the DNA substrate processing, its recruitment to conjugative pores, and the subsequent DNA translocation reactions. More relevant to this work, how conjugation is integrated with plasmid maintenance functions such as replication and segregation is still poorly understood.

The low-copy number conjugative plasmid R388 is the prototype of the IncW family. It represents the smallest broad host range conjugative plasmid [10], [11]. Its mechanism of segregation is presently unknown, and no *par* system was annotated in its DNA sequence [12]. R388 carries a cluster of two operons, which are transcribed divergently from the region containing *oriT*. One contains the MOB genes, and the other includes a cluster of three genes, *stbA*, *stbB* and *stbC*, whose functions have not been analyzed previously. Their relative position to *oriT* makes these three genes the first to enter the recipient cell during plasmid conjugation. Here, we investigated the role of the *stbABC* operon in plasmid R388 transfer and stability in *E. coli*. By using a fluorescent protein to tag plasmid molecules, we found that R388 plasmid foci, most of which contain a single copy of the plasmid, are evenly distributed within *E. coli* cells. In contrast, a derivative of R388 lacking *stbA* mislocalized as clusters at the cell poles or at the cell center, and correlated with plasmid instability. In addition, we show that StbB, a putative ATPase protein, is strictly required for R388 conjugative transfer and the occurrence of plasmid foci close to the membrane cell poles. Taken together, these results suggest that *stbA* and *stbB* constitute a balancing system that integrates plasmid conjugation with the functions that ensure the efficient vertical transmission of the plasmid.

## Results

### Comparative genomics of the *stb* operon

**Figure 1:**
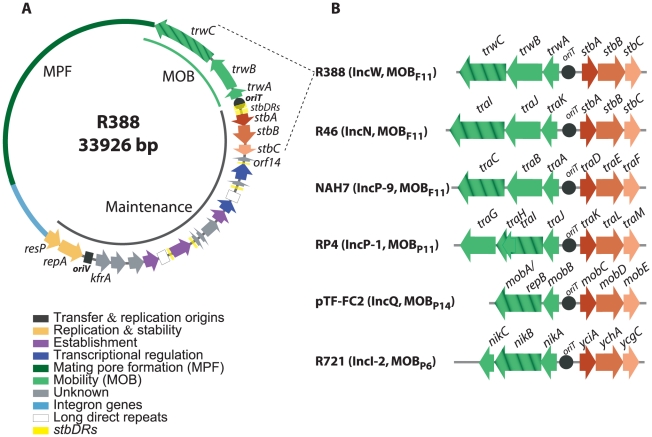
The *stbABC* operon. A: genetic organization of plasmid R388. ORFs and other sequence features are depicted in different colors according to the legend on the left, after assignment to functional modules based on database similarity and/or available experimental data [12]. The R388 genome is compact and organized in two major modules, one devoted to general maintenance functions and one devoted to conjugation (MOB, Mobility genes; MPF, Mating Pore Formation). *stbDRs*, located around the promoter region of the *stbABC* operon and consisting of two sets of five direct repeats of a 9 bp DNA sequence spaced by two nucleotides, are indicated. This 9 bp DNA sequence is also present in number of two or three in three other promoters of the maintenance region. B: Synteny conservation between the MOB-*Stb* region of R388 and those of other plasmids. Hatched arrows indicate the relaxase gene. Plasmid GenBank accession numbers: R388: BR000038, R46: NC_003292, NAH7: NC_007926, RP4: L27758, pTF-FC2: M57717.1, R721: NC_002525.

To study the role of the three *stb* genes in plasmid R388, we first analyzed the *stb* operon by protein sequence comparison. We found that the most conserved protein is StbB, whereas StbA is poorly conserved. Besides, StbC is an orphan protein, without significant homologs in any other system. We thus used R388 *stbB* gene as template to search for homology. StbB homologs were usually included in operons of three genes at the leading region of conjugative plasmids of MOB_F11_, MOB_P11_, MOB_P6_ and of mobilizable plasmids MOB_P13/P14_, belonging to several Inc groups (IncW, IncN, IncP-1, IncP-9, IncQ and IncI-2). Synteny conservation of the *stb* and MOB regions of representative plasmid groups is shown in Figure 1B. There was a neat bias for the presence of *stbB*-like genes in conjugative plasmids that carried an MPF_T_ T4SS. In addition, an Stb-system was also found in some groups of mobilizable plasmids. In all cases, the *stb* genes were located at the 3′side of the nicked strand and are thus the first to enter the recipient cell during plasmid conjugation.

Alignment of StbB and homologs from each MOB group showed that they shared a deviant Walker A nucleotide triphosphate-binding motif (Figure S1A), also found in the ParA/Soj/MinD superfamily of ATPases [13]. Members of this superfamily include ParA, required for accurate chromosomal and plasmid DNA partitioning, MinD required for correct placement of the septa during cell division, and Soj, which plays a role in chromosome compaction required for nucleoid partition (reviewed in [3], [14], [15]). *parA* and *soj* genes are found in their respective operons adjacent to a second gene, *parB* and *spo0J* respectively, which encodes a DNA-binding protein. However, no homology of StbA to ParB-like partition proteins was detected. On the other hand, R388 StbA showed significant homology to the TraD protein of plasmid NAH7. Iterative PSI-BLAST also returned similarly located proteins in MOB_P11_, MOB_P6_ and MOB_P13/P14_ plasmids (including StbA_R46, TraK_RP4, MobC_pTC-FC2, and YciA_R721) after seven iterations. Alignment of R388 StbA and several similarly located proteins is shown in Figure S2.

According to the sequence of their StbB-like proteins, plasmids can be phylogenetically assorted in two large groups. The first group includes MOB_P11_, MOB_P6_ and MOBP_13/14_ plasmids, that encode StbB-like proteins (including TraL_RP4 or MobD_pTF-FC2) containing the classical motif KGGXXK[T/S] found in other ParA/Soj/MinD ATPases [13]. R388 plasmid belongs to the second group together with other MOB_F11_ and MOB_P6_ plasmids. These encode StbB-like proteins that contain the slightly divergent motif SGXXGK[T/S]. MOB_P6_ plasmids are pervasive in both groups, perhaps suggesting that they were the first in which the Stb-system was installed. Modeling the structure of StbB using the 3D structure of Soj (PDB ID: 2BEK) as template predicts that the dimerization role of the signature lysine K15 in Soj, [16] could be performed by other polar residue like serine S9 in R388-StbB (Figure S1B).

### StbA and StbB are involved in plasmid R388 conjugative transfer

**Figure 2:**
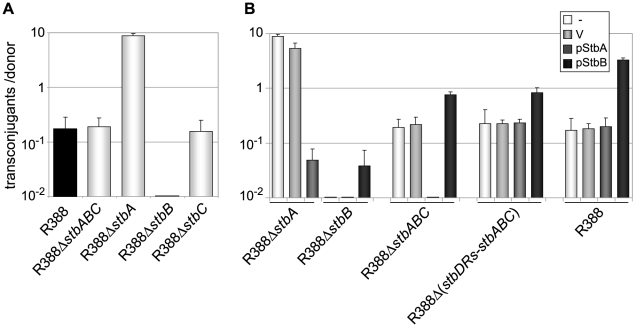
Conjugation frequencies of plasmid R388 derivatives. Plasmids contained in the different donor strains are indicated on the X axis. The figures shown represent the mean and standard deviation of at least four independent assays. A: Donor strains contain the R388 derivatives shown; B: Complementation analysis. Donor strains contain the same R388 derivatives plus a helper complementing plasmid, as shown (V  =  empty vector).

To investigate whether the *stb* operon was involved in R388 transfer, as suggested by its positional conservation relative to the MOB region, we first generated a derivative of R388 deleted of the entire *stb* operon, R388Δ*stbABC*. The method of Datsenko and Wanner [17] was used to replace the *stb* region by a DNA fragment conferring resistance to kanamycin. Mating experiments were carried out under appropriate conditions to avoid indirect effects due to plasmid instability (see below and Materials and Methods). As shown in Figure 2A, deletion of *stb* did not result in any noticeable effect on transfer frequencies compared to the wild-type plasmid R388 (R388). These results, which are consistent with previous data [18], could in principle suggest that the *stb* operon was not involved in R388 transfer. We nevertheless examined the effect of deleting each of the three genes of the *stb* operon independently. We constructed three R388 derivatives, R388Δ*stbA*, R388Δ*stbB*, and R388Δ*stbC*, which lack *stbA*, *stbB*, and *stbC*, respectively, and measured their transfer frequencies (Figure 2A). Surprisingly, R388Δ*stbA* was transferred at a frequency approximately 50-fold higher than R388, suggesting that StbA inhibits R388 conjugative transfer. In contrast, deletion of *stbB* resulted in a complete block of conjugation (transfer frequency <10E-9, Figure 2A), indicating that StbB is required for R388 conjugation. The transfer frequency of R388Δ*stbC* was comparable to that of R388, which indicated that StbC had no significant role in R388 conjugative transfer. Since deletion of the entire operon did not modify the conjugation frequencies, we concluded that StbB is required for conjugation only in the presence of StbA, which in turn, inhibits conjugation. StbA and StbB thus appear to have antagonistic effects in conjugation.

To further examine the interactions between the different functions of *stb* genes, we carried out a complementation analysis. We constructed plasmids carrying either the *stbA* or *stbB* genes, driven by the P*lac* promoter and controlled by a *lacI ^q^* gene present on the same plasmid (pStbA and pStbB, respectively, Table S1). pStbA and pStbB were introduced in donor cells by transformation, and mating experiments were carried out. Results are shown in Figure 2B. Supplying StbA in *trans* in donor cells harboring plasmid R388Δ*stbA* led to a 100-fold reduction in conjugation, which corresponded to a frequency comparable to that of R388. This result also indicated that the *stbB* gene was adequately expressed in plasmid R388Δ*stbA* (that is, the *stbA* deletion did not cause a polar effect). Supplying StbB in *trans* in donor cells containing plasmid R388Δ*stbB* led to restoration of the transfer frequencies to wt level.

Providing StbA in *trans* in donor cells harboring plasmid R388Δ*stbABC* abolished transfer (Figure 2B), further demonstrating that StbB is required for conjugative transfer only when StbA is present, and that in turn StbA inhibits conjugation in the absence of StbB. Besides, supplying StbB in *trans* to plasmid R388Δ*stbABC* resulted in an increase of conjugation frequency of approximately 4-fold. This indicated that StbB stimulates conjugative transfer in the absence of StbA. We next introduced either pStbA or pStbB in donor cells containing plasmid R388 to analyse the effects of overexpressing the corresponding *stb* gene. Supplying StbA in *trans* had no effect, while supplying StbB in *trans* led to enhanced transfer frequencies to a level similar to that of plasmid R388Δ*stbA* (Figure 2B). This indicated that StbB stimulates conjugation also when StbA is present.

Taken together, the results shown in Figure 2 demonstrate that StbA and StbB, but not StbC, are involved in R388 conjugation in *E. coli*, and that their activities are functionally connected. StbB was strictly required for conjugation only when StbA was present. Besides, StbA prevented R388 conjugative transfer, whereas StbB stimulated R388 conjugative transfer, suggesting that StbA and StbB have balancing/compensatory effect to control conjugation.

### StbA is required for plasmid inheritance

**Figure 3:**
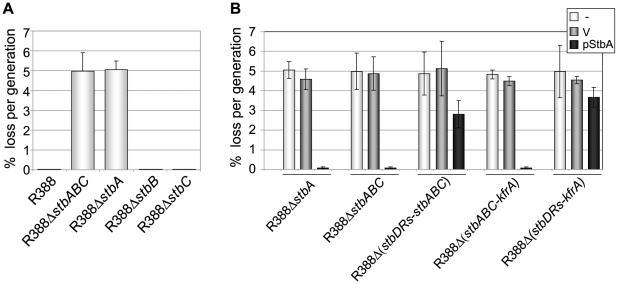
Stability of plasmid R388 derivatives. The stability of a series of derivatives of R388 was measured as the rate of loss per generation from strain LN2666 as described in Materials and Methods. Error bars show standard deviations calculated from at least four repeats of each assay. A: loss rates of R388 derivatives; B: loss rates of R388 derivatives in the presence of StbA. The loss rates of R388 derivative plasmids were measured when coresident with a second replicon (a p15A-derivative) as indicated in the top right panel (-, none; V, vector; pStbA, carrying *stbA*).

Previous studies showed that the *stb* genes of the IncN plasmid R46 are required for stable plasmid inheritance in recombination-proficient but not in *recA* strains [19]. To examine whether R388 *stb* operon plays a role in plasmid R388 inheritance, plasmid R388Δ*stbABC* was subjected to stability studies in LN2666 (*recA+*) and in FC232 (LN2666 *recA*) strains. As we obtained similar results in both genetic backgrounds, only the results with LN2666 strain are presented in Figure 3. *E.coli* cells carrying R388Δ*stbABC* were grown in serial cultures in nonselective medium, and plasmid loss rates were measured by plating out every 20 generations to determine the proportion of cells retaining the plasmid (Materials and Methods).

Plasmid R388 was stably maintained in progeny cells and 100% of the cells retained the plasmid after 80 generations (Figure 3A). Deletion of the entire *stb* operon led to a significant stability defect with a rate of loss of 5% per generation. We next examined the effects of deletion of each *stb* gene on plasmid stability. R388Δ*stb*A showed a plasmid loss rate similar to that of R388Δ*stbABC* (5.1% loss per generation). In contrast, R388Δ*stbB* and R388Δ*stbC* loss rates were close to zero (10^−2^% and 8.10^−3^% per generation, respectively; Figure 3A). The stability defect of R388Δ*stbABC* was thus fundamentally due to the absence of *stbA*.

Complementation of R388Δ*stbABC* and R388Δ*stb*A with the StbA-producing plasmid pStbA decreased their loss frequency to 0.07% and 0.08%, respectively (Figure 3A). This result confirmed that StbA is required for the stable inheritance of R388 and showed that it acts *in trans*. We then addressed the question of whether the instability of plasmids R388Δ*stbABC* and R388Δ*stbA* was due to a decrease in their plasmid copy number per cell when compared to R388. However, the average number of copies per chromosome of both plasmids, as determined by real-time qPCR (Materials and Methods), were found to be similar to that of R388 (R388, 3.8±1.0, R388Δ*stbABC*, 4.5±1.2 and R388Δ*stbA*, 4.1±0.9). This result strongly suggested that the instability of R388Δ*stbA* and R388Δ*stbABC* was due to a defect in plasmid segregation.

### StbA operates in *trans* on *cis*-acting *stbDRs*

The above results indicated that StbA is involved both in the stability and in conjugative transfer of plasmid R388, raising the question of how StbA interacts with R388 to perform such functions. The StbA homolog TraK_RP4 was shown to bind specifically to a DNA sequence containing the region between the *nic* site and the *traK* gene [20]. We thus explored the role of the upstream region of *stb*, which contains two sets of five direct repeats of a DNA consensus sequence 5′ TTGCATCAT (the *stbDRs*, Figure 1).

StbA protein was purified on a Ni-agarose resin as a StbA-His_6_-tagged protein (Materials and Methods). Incubation of a DNA fragment containing a complete set of *stbDRs* with increasing quantities of StbA and an excess of nonspecific competitor DNA gave rise to retarded species (Figure S3). Thus, StbA bound specifically *stbDRs* containing DNA *in vitro*.

To examine the role of the *stbDRs in vivo*, we constructed a derivative of plasmid R388Δ*stbABC* carrying a larger deletion which included the *stbDRs* (R388Δ*stbDRs-stbABC*). While providing StbA in *trans* to plasmid R388Δ*stbABC* abolished transfer (Figure 2B), StbA had no effect on conjugation of R388Δ (*stbDRs-stbABC*) (Figure 2B), showing that the role of StbA in R388 conjugation is mediated by its binding to the *stbDRs*: if StbA does not bind the *DRs*, StbB is not required for conjugation. Besides, providing StbB *in trans* to R388Δ*stbABC*, as well as to R388Δ (*stbDRs-stbABC*) results in 4-fold increase in transfer frequencies (Figure 2B), suggesting that the role of StbB does not depend on the presence of the *stbDRs*.

We next examined the role of the *stbDRs* in plasmid stability. R388Δ (*stbDRs*-*stbABC*) showed the same instability phenotype as R388Δ*stbABC* (Figure 3B). Providing StbA *in trans*, led to an almost complete reduction of R388Δ*stbABC* plasmid instability (rate of loss of 0.07% per generation). Furthermore, providing StbA *in trans* had only a partial stabilization effect on the R388Δ (*stbDRs*-*stbABC*) plasmid (rate of loss of 2.8% per generation). We concluded that, as for conjugation, the role of StbA in R388 stability is fundamentally mediated by its binding to the *stbDRs*.

Analysis of R388 genome showed that the 9-bp motif specific of the *stbDRs* is found in four other promoters within the establishment region of R388 (Figure 1; [12]). To examine whether these potential StbA binding sites sequences could have a role in R388 stability, we generated a series of plasmids carrying several deletions of this region. We found that, provided that the *stb* operon was preserved, the deletion of a DNA fragment ranging from *orf14* to *kfrA* did not affect plasmid stability at all (R388Δ (*orf14-kfrA*), rate of loss of 0%, Figure 1A, Table S1). This indicated that this region does not contain genes required for R388 stability and demonstrated that within the establishment region, only the *stb* genes are required for R388 stability. As expected, a plasmid lacking the entire establishment region (i.e. from *stbA* to *kfrA* gene, see Figure 1, R388Δ (*stbABC*-*kfrA*)) was not stably maintained (rate of loss of 5%, Figure 3B). Supplying StbA *in trans* led to the stabilization of such a plasmid carrying the *stbDRs* region (R388Δ (*stbABC*-*kfrA*), Figure 3B). As expected, this stabilisation effect was dependent on the *stbDRs* since the equivalent plasmid lacking the *stbDRs*, R388Δ (*stbDRs*-*kfrA*), remained highly unstable upon StbA production (3.7% of loss per generation). We concluded that the potential StbA binding sites located outside the *stbDRs* are not sufficient to support StbA-dependent plasmid stabilization.

### Localization of R388 DNA molecules in *E. coli* living cells

**Figure 4:**
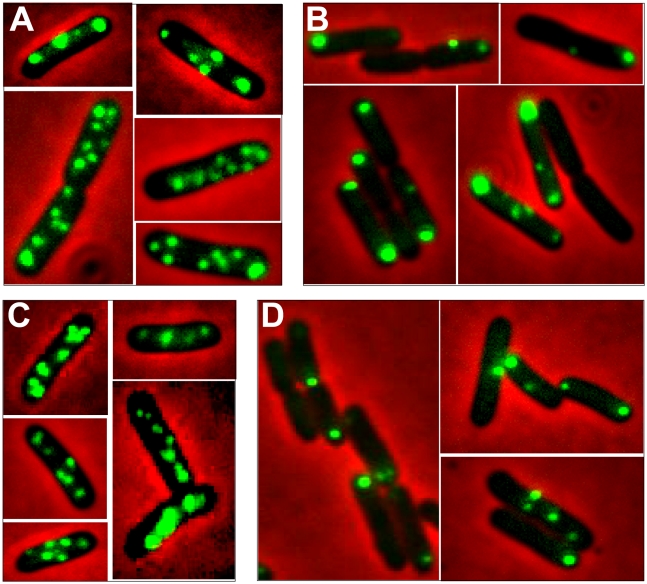
Localization of plasmid R388 and derivatives in live *E. coli* cells using a *parS*/GFP-ParB system. LN2666 cells containing R388*parS* and expressing GFP- Δ30ParB from plasmid pALA2705 [21] were grown in M9 medium supplemented with glucose and casamino acids at 30¼C, without IPTG induction, as described in Materials and Methods. The fluorescence pictures were merged with the phase contrast pictures. GFP-tagged plasmids appear as green foci. Representative images are shown. A: R388; B: R388Δ*stbA*; *C*: R388Δ*stbB*; D: R388Δ*stbABC*.

To get a deeper insight into R388 segregation, we undertook a cellular localization study of plasmid R388 using the *parS*/GFP-ParB system [21]. The *parS* site of bacteriophage P1 was inserted into R388 plasmid DNA as a *parS*-chloramphenicol (*parS*-*Cm*) cassette. This allowed visualization of the cellular localization of the resulting plasmid R388*parS* (R388, Table S1) in live *E. coli* cells expressing the green fluorescent marker protein GFP-Δ30ParB from a second plasmid (pALA2705; [21]; Materials and Methods). We used two different R388*parS* plasmids (R388*parS1* and R388*parS2*), in which the P1 *parS* site had been inserted in two different intergenic regions R388 and obtained comparable results (Materials and Methods; data not shown). Figure 4A shows representative fluorescence images of cells containing R388*parS1* plasmid. Discrete foci could be visualized without IPTG induction, at the basal level of expression of the fluorescent GFP-Δ30ParB from the *lac* promoter [22]. Under these conditions, neither the *parS* insertions into R388, nor the expression of the GFP-Δ30ParB protein had a noticeable effect on R388*parS* plasmid stability and conjugative transfer (plasmid loss rate <0.01%; conjugative transfer frequency 2.10^−1^/donor).

**Figure 5:**
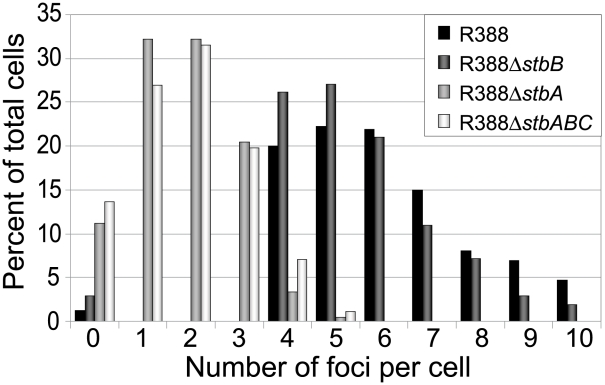
Comparison of the relative number of foci in cells harbouring plasmid R388 or derivatives. Percentage of total cells (Y axis) is plotted against the number of GFP foci per cell (X axis) for each R388 derivative as indicated in the top right panel.

In the conditions used, more than 98% of the cells analyzed contained GFP-foci, showing that the efficiency of focus detection was high. There were 4 to 10 foci per cell and the average number of foci per cell increased with cell size (data not shown). A majority of the cells (64%) had 4 to 6 foci, and about 34% had 7 to 10 foci (Figure 5, R388). The population average was approximately 6 foci per cell, with most small cells harboring 4 foci and longest ones harboring 8 to 10 foci. The number of foci per cell thus roughly corresponded to the copy number of R388 as calculated by qPCR, suggesting that most observed foci contained a single copy of the plasmid.

**Figure 6:**
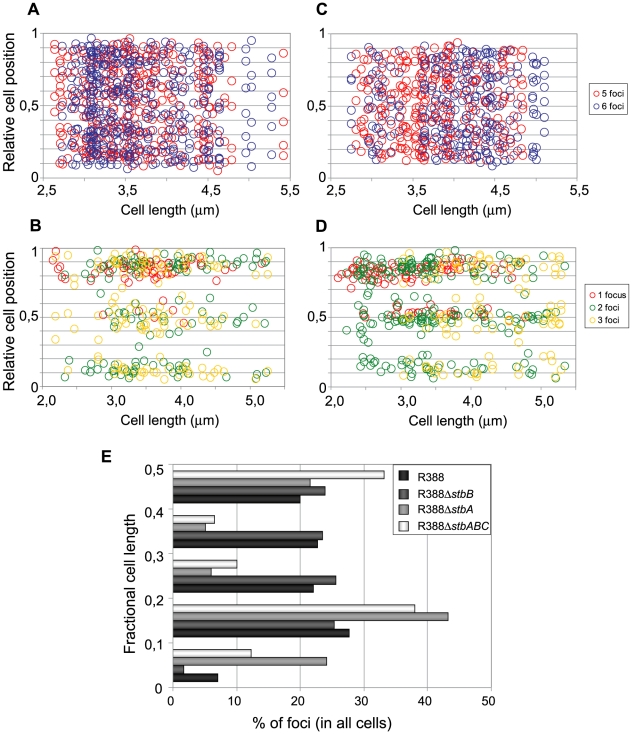
Subcellular distribution of GFP-tagged R388 derivatives in live *E. coli* cells. Cultures were grown as described in legend of Figure 4 and in Materials and Methods. A to D: Relative positions of the GFP foci along the long axis as a function of cell length in at least 200 cells. Foci are represented as color points as indicated in the right panels. A: R388 (n = 320); B: R388Δ*stbA* (n = 205); C: R388Δ*stbB* (n = 210); D: R388Δ*stbABC* (n = 309). n, number of analysed cells. E: Distribution of GFP foci within the different fractions of cell length. The distance of foci to the closest cell pole was measured and sampled into five equal sections of cell length from the pole to mid-cell.

To determine the subcellular position of R388 plasmids, the distance from one cell pole to each focus was measured and plotted as a function of the cell length. For the sake of clarity, only distributions of foci in cells with 5 and 6 foci are presented in Figure 6A. Foci were broadly located and we observed no evidence for preferential positioning at the cell centre or at the ¼ and ¾ cell length positions. However, foci distribution was not random. Indeed, the proportions of cells containing at least one focus in each cell quarter were significantely different from those expected for a random distribution (Observed/expected for a random distribution: 45%/9,4% for 4-foci cells; 86%/23,4% for 5-foci cells; 89%/38,1% for 6-foci cells; p-values <10^−4^ in all cases using the χ^2^ test). Thus, foci appeared to be evenly distributed along the cells, suggesting a mechanism of active distribution of R388 plasmid copies.

To obtain a global view of foci assortment, we counted the number of foci located within five fractions of half-cells length (Figure 6E). The majority of foci were positioned equally within the four central slices of cells (from 0.1 to 0.5 fractional cell length). However, 7% of foci were located within the most polar region (from 0 to 0.1 fractional cell length, Figure 6A and 6E), showing that the broad distribution of foci extended to the cell poles.

### R388 subcellular localization depends on StbA

We next observed the subcellular localization of the unstable R388Δ*stbA* plasmid, using the same *parS*/ParB-GFP system and conditions described above. Under these conditions, neither the *parS* insertions into R388, nor the expression of the GFP-Δ30ParB protein had a noticeable effect on R388*parS* plasmid stability and conjugative transfer (plasmid loss rate <0.01%; conjugative transfer frequency 2.10^−1^/donor). Representative images are shown in Figure 4B. About 11% of the cells were devoid of fluorescent focus (Figure 5), a value consistent with the degree of instability of the plasmid. A majority of cells showed a number of foci in the range of 1 to 3 (85%, Figure 5). The population average was 2 foci per cell, which is approximately 3-fold lower than R388. As mentioned above, the copy number of R388Δ*stbA* was found to be similar to that of R388, implying that most R388Δ*stbA* foci contained 2 or 3 plasmid copies. Thus, the decrease in number of foci is most likely due to plasmid clustering.

The subcellular distribution of foci in cells harboring R388Δ*stbA* is shown in Figure 6B and 6E. In cells with only one focus, the single focus was primarily in the polar region (91% of foci located from 0 to 0.2 fractional cell length). All cells having two or three foci had at least one polar focus. In cells having two foci, the other focus was localized mainly at the opposite polar region (48%) or in the cell center (38%). In cells having 3 foci, a majority had one focus at each pole and the other at the cell center (55%) or one focus at a pole and the two others at the cell center (43%). All in all, approximately 33% of the focus-containing cells contained all foci in one side of the cell and no focus close to the center, i.e., they are cells that would give rise to plasmid-free cells if the cell divided at the mid-position without any change in plasmid number or position. The location of plasmid foci relative to the nucleoid was determined by visualizing cells stained with DAPI (Figure S4). Fluorescence foci were mainly localized in nucleoid-free areas that were not occupied by the chromosomal DNA, either at the cell poles or at the cell center in the cytosol space between two nucleoids in dividing cells. Therefore, subcellular distribution of the unstable R388Δ*stbA* plasmid is markedly different from that of the stable R388 plasmid carrying *stbA*, and correlated with its instability in *E. coli* (see above).

### StbB is required for R388 localization at the cell poles

To assay whether StbB had a role in R388 localization, we observed R388Δ*stbB*-harbouring cells (Figure 4C and Figure S4). The distribution of foci was almost identical to that of R388. More than 97% of the cells contained GFP-foci, and had focus numbers in the range of 4 to 10 (Figure 5), with a population average of 5.6 foci per cell. The mean number of R388Δ*stbB* molecules per cell was found to be 4.0±1.1, showing that, similarly to what was observed with R388, most foci contain a single molecule of the plasmid.

Figure 6C shows the subcellular positioning of R388Δ*stbB* plasmid molecules in cells containing 5 and 6 foci. R388 deleted of *stbB* was evenly distributed within the cell with the exception of the most polar region (from 0 to 0,1 fractional cell), which appeared to contain less foci than R388. Comparison of R388Δ*stbB* and R388 foci distributions within the five fractions of half-cells length (Figure 6E) using the χ^2^ test revealed that they were indeed significantly different (χ^2^ = 61.1 corresponding to a p-value <10^−4^). This difference can be attributed to a different polar localization of R388 and R388Δ*stbB* since the distribution of these plasmids within the three central slices of the half-cell (from 0.2 to 0.5) were not significantly different (p-value  = 0.18). We concluded that StbB is required for the localization of a fraction of R388 copies towards the cell poles. Without StbB, R388 molecules are excluded from the poles.

To further investigate the role of StbB in intracellular positioning, we compared the localization of the R388Δ*stbA* and R388Δ*stbABC* plasmids. As in the case of R388Δ*stbA* (see above), R388Δ*stbABC* formed 1 to 5 fluorescent foci per cell, with a majority (78%) of cells containing 1 to 3 foci (Figure 4D and Figure 5), and showing a strong bias for location at the center and cell poles (Figure 6D). However, R388Δ*stbABC* and R388Δ*stbA* foci distributions were found to be significantly different (p-value <10^−4^). As illustrated in Figure 6E, this difference mainly relied on the proportion of foci within the most polar region (from 0 to 0.1 fractional cell length; R388Δ*stbABC*, 12%, compared to R388Δ*stbA*, 24%) and at the center (R388Δ*stbABC*, 33%, compared to R388Δ*stbA*, 22%). Thus, StbB is required for the localization of R388 at the pole and midcell positions in both the presence and absence of StbA.

## Discussion

**Figure 7:**
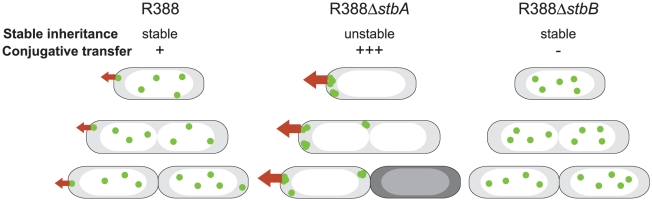
A schematic model to explain the behavior of *stb* mutants of plasmid R388. The model explains the correlation between the plasmid subcellular localization and its properties during cell division. Green closed circles indicate plasmid molecules. Open and shaded regions indicate nucleoid and cytosol spaces, respectively. Red arrows represent conjugative transfer capacity. A: DNA molecules of plasmid R388 are evenly distributed both within nucleoid and cytosol spaces. Septum forms at midcell. Therefore, each resulting newborn cell contains plasmid copies. Localization of plasmid molecules close to the cell membrane is correlated with R388 capacity to undergo conjugative transfer. B: DNA molecules of plasmid R388Δ*stbA* are exclusively localized in cytosol spaces towards the cell poles and the cell center. Mislocalization is correlated with plasmid instability, since cells containing all foci in one side of the cell give rise to plasmid-free cells (dark), and high conjugation frequency, since there are more plasmid copies at the poles. C: DNA molecules of plasmid R388Δ*stbB* are distributed in nucleoid but not in cytosol spaces. This mislocalization is correlated with a defect in conjugative transfer.

In this study, we show that protein StbA is strictly required for stability and intracellular positioning of plasmid R388 in *E. coli*. We found that fluorescent foci of R388, most of which contain a single copy of the plasmid, are evenly distributed along the cell, with no evidence for preferential localization. This is in contrast with other low-copy number plasmids, such as mini-F, mini-P1, R27 and RK2 plasmids, which were reported to localize as clusters at the ¼–¾ or midcell positions [21], [23]–[26]. In these cases, duplication of the central focus is presumed to represent active partition of plasmid copies. However, it has been recently shown for mini-P1 plasmid that more than two foci are present in most conditions, and that the behavior of foci is more dynamic than previously reported [27]. This model of segregation of mini-P1 is more consistent with the even distribution of R388 copies that we have observed. We thus hypothesize that, as proposed for mini-P1, R388 copies segregate as single units and distribute into a dynamic and evenly spaced pattern along the cell to ensure a proper distribution of the plasmid copies at cell division. Moreover, in contrast to non-conjugative mini-F and mini-P1 plasmids, which were reported to be contained within the nucleoid region [21], [23], [25], [27], a significant fraction of R388 foci are found at the extreme cell ends. This observation may reflect plasmid R388 ability to undertake conjugative transfer. In agreement with this, we have recently shown that R388 coupling protein TrwB localizes to the cell poles (data not shown). Besides, it has been reported that the T4SS apparatus of *Agrobacterium tumefaciens* and of plasmid pCW3 from *Clostridium perfringens*, assemble at the cell poles [28]–[31]. In contrast to the even distribution of R388, R388Δ*stbA* plasmid foci clustered at the cell poles or at the cell center, in nucleoid-free areas. Mislocalized plasmid clusters appear to be the main cause of instability, as they are not adequately distributed in cellular spaces corresponding to future daughter cells (Figure 7). Mislocalized plasmid clusters were reported in derivatives of plasmids mini-F, mini-P1, R27, and R1 in which their Par regions were inactivated [25], [32]–[34]. In these cases, plasmid *foci* appeared distributed randomly in nucleoid-free spaces. This similarity suggests that StbA acts as a partition system. Indeed, the *stb* operon shares many characteristics with Par systems implicated in plasmid and chromosome partitioning. StbA is a DNA-binding protein which binds a *cis*-acting sequence (it is thus a ParB-like protein), the *stbDRs*, and StbB is a putative motor protein harboring Walker-type ATPase motifs (thus a ParA-like protein). However, and in contrast to ParA-like counterparts, StbB is not required for R388 stability, suggesting that the StbAB system does not constitute a typical ParAB system. StbB may either have no role in R388 maintenance or may be replaced by an equivalent cellular function when inactivated. Alternatively, R388 segregation may not need to involve an active motor. In this view, the StbA-*stbDRs* complex may be used to pair plasmid molecules with the host chromosome, ensuring an even distribution of R388 copies along the nucleoid length by an unknown mechanism.

Besides, we demonstrate that, although deletion of the entire *stb* operon does not affect conjugation, StbA and StbB, but not StbC are involved in R388 conjugation. Deletion of *stbA* results in an enhanced frequency of conjugation, while deletion of *stbB* leads to a conjugation defect, indicating that StbA and StbB have opposite but connected effects. As explained above, our results further suggest that these conjugation defects are a consequence of variations in the intracellular positioning of plasmid DNA. Whatever the way StbA promotes R388 segregation, the associated localization is certainly not convenient for maximal conjugation frequency, since StbA inactivation, associated with plasmid localization at the cell poles, strongly enhances conjugation. In addition, R388Δ*stbB* conjugation defect correlates with the absence plasmid foci at the cell polar membrane (Figure 7). We thus assume that the role of StbB is either to counteract StbA by locating some plasmid copies at the polar conjugal transport site, thereby allowing conjugation to occur, or StbA may modulate the activity of StbB and/or delocalize the plasmid copies.

The way the relaxosomal complex is transferred to conjugative pores remains unknown. It was previously suggested that *A. tumefaciens* VirC1 protein, which belongs to the ParA and Soj/MinD ATPases family, spatially positions the relaxosome at the cell pole to coordinate substrate-T4SS docking [35]. StbB also shares features with ParA/Soj/MinD ATPases (Figure 2B). These proteins are though to employ a principle of dynamic oscillation between specific surfaces such as membrane or bacterial chromosome to explore and mark the cellular environment [4]. Several models for the action of the Walker partition ATPases have been proposed. Formation of dynamically unstable filaments in a nucleotide-dependent manner was suggested following the example of actin-type partition ATPases [4]. Such cytomotive filaments could achieve partitioning by pushing plasmids attached to growing filaments, or by pulling plasmids attached to retracting filaments and there has been some evidence for both modes of action [36], [37]. Alternatively, a diffusion ratchet model was proposed (Vecchiareli et al., 2010). In this case, the motive force for plasmid positionning does not rely on the ParA ATPase polymerization, but instead is directed toward regions of high ParA concentration. These models are all consistent with our current data and such dynamic modes of action constitute appealing mechanisms for how StbB might recruit R388 molecules from a cytosolic pool to the membrane for conjugative transfer. Since inactivation of either the coupling protein (TrwB) or the relaxase (TrwC) did not affect plasmid R388 cellular localization (data not shown), the StbB-dependent mechanism of transport of R388 molecules to the cell membrane is neither associated with relaxosome formation, nor it requires either the coupling protein or cleavage at *oriT*. StbB may interact either with plasmid DNA or with StbA bound to DNA to form nucleoprotein complexes analogous to the ParA/ParB/*parS* partitioning complex, but linked to conjugative DNA processing. We are presently investigating the detailed molecular mechanisms by which StbB interacts with R388 molecules to recruit them to the cell membrane prior to transfer.

Our comparative genomics studies showed the conservation of synteny of three genes, of which the second gene is the most conserved, at the leading region of conjugative plasmids of mobility groups MOB_F11_, MOB_P11_, MOB_P6_, indicating that the *stbABC* operon is widespread among plasmids. Moreover, the *stb* operon is apparently linked to MPF_T_ T4SS systems, although not exclusively, as it is also carried by mobilizable plasmids of the MOB_P13/P14_ group. It remains to be explored if these plasmids require a MPF_T_-type T4SS for conjugative transfer. Synteny conservation may reflect a requirement for *stb* in plasmids carrying such conjugation machinery under natural conditions. This is supported by our observation that the presence of R388 transfer region leads to instability of a pBR322-derivative plasmid containing it (data not shown).

In summary, we present experimental evidence that the StbAB system constitutes an atypical plasmid stabilization system intimately linked to conjugative transfer. On one hand, StbA is strictly required for plasmid R388 stability. Its inactivation results in mislocalization of R388 copies towards the cell poles, which is correlated with a significant increase in transfer frequencies (Figure 7). On the other hand, StbB is necessary for conjugative transfer only in the presence of StbA. Its inactivation leads to conjugation defect, which is associated with the absence of plasmid molecules at the extreme cell poles (Figure 7). Our results thus suggest that the StbAB system may act as a molecular scale between two possible transmission modes of plasmid R388: vertical transmission by faithful segregation to daughter cells and horizontal transmission by conjugative transfer to a different cell (Figure 7). It would seem that an active conjugation system provokes plasmid instability per se, which could only be counterbalanced by Stb or an analogous stability system. In this case, it remains to be investigated if other plasmids use functionally analogous but phylogenetically unrelated systems to balance their propagation and vegetative modes. This is, to our knowledge, the first report of a system involved in the reconciliation of these two cellular processes.

## Materials and Methods

For bacterial strains, plasmid constructions and oligonucleotides, see Text S1 and Tables S1 and S2.

### Conjugation tests

For mating experiments, donor (LN2666; [38]) and recipient (BW27783; [39]) strains were grown overnight from single colonies in LB medium at 37°C with appropriate antibiotics. After washing, 50 µl of donor cells were mixed with 800 µl of recipient cells, the mixture centrifuged for 1 min, resuspended in 10 µl LB medium and cells placed onto a GS Millipore Filter (0.22 µm pore size) on a LB-agar plate at 37°C for 20 min, which corresponds to a period shorter than the generation time to limit indirect effects due to plasmid instability. Bacteria were then washed from the filter, diluted in 2 ml LB medium and serial dilutions plated on selective media. Conjugation frequencies were expressed as the number of transconjugants per donor cell. When providing plasmids pStbA or pStbB (Table S1), the amount of protein StbA or StbB produced was found to be sufficient to restore the wt phenotype without the need to induce their expression with IPTG.

### Plasmid stability assays

Single colonies of LN2666 or DH5α strains (Table S1) containing R388 or a derivative of it were used to inoculate LB containing selective antibiotics, and the cultures were incubated overnight at 37°C. For each strain, stability experiments were performed at least four times, starting from separate colonies. 48.8 µl of a 10-fold dilution of overnight cultures were transferred to 5 ml LB, containing streptomycin (300 µg/ml) but lacking the antibiotic selective for the plasmid, and grown for 12 h, i.e. for 10 generations. These freshly inoculated cultures constituted time point zero. From then on, 48.8 µl of a 10-fold dilution of the full-grown cultures was transferred every 12 h to fresh 5 ml LB and incubated at 37°C to reach a total of 80 generation times. Each 12 h, the cultures were also diluted and plated onto LB plates. Determining the fraction of plasmid free cells in the population was done by replica-picking 100 randomly chosen colonies per culture from the LB plates onto LB plates containing the appropriate selective antibiotics, and scoring the proportion of colonies with a given resistance. The percentage of plasmid loss per generation was calculated as described in Yates et al., 1999 [40].

### Live cell fluorescence microscopy

A derivative of strain LN2666 (*recA+*) containing plasmid pALA2705 (Ap; Table S1), which produces the fluorescent GFP-Δ30ParB protein [21], was transformed with DNA of plasmid R388::*parS-Cm* or one of its derivatives. Neither the *parS* insertions into R388 (or *stb* derivatives listed in Table S1), nor the expression of the GFP-Δ30ParB protein had a noticeable effect on the corresponding R388*parS* plasmid stability and conjugative transfer (data not shown). Single-colony isolates were grown overnight in M9 medium supplemented with 0.2% casamino acids, 0.4% glucose, 2.0 µg/ml thiamine, 20 µg/ml leucine and 20 µg/ml thymine, (suppl. with Ap, Cm) at 30°C. Cultures were then diluted 1/100 in the same medium and grown at 30°C to OD_600_ of 0.8. With these constructions, foci could be adequately visualized without the need to induce expression of the fluorescent GFP-Δ30ParB protein [27]. When needed, DAPI stain (1 µg/ml; Molecular Probes) was added to the culture for 20 min to label DNA. Suspensions of growing cells were directly deposited on glass slides covered by a layer of 1% agarose containing the same growth medium and examined by phase-contrast and fluorescence microscopy. Images were captured with an inverted Olympus X81 microscope equipped with a 100x oil-immersion Olympus lens (N.A. of 1.3) and a Roper Coolsnap CCD camera, using Metamorph software. Cell length and focus position was measured manually using ImageJ. Each strain was examined in at least four independent experiments with similar results. At least 200 cells were inspected for each experimental observation.

## Supporting Information

Figure S1 StbB proteins contain a putative deviant Walker A ATP-binding motif (P-loop). A: Sequence alignment of R388 StbB protein and homologs from representative plasmid families. The sequence and the numbering highlighted above the alignment are from the crystal structure of Soj (PDB ID: 2BEK). The secondary structure elements of Soj are also shown above the alignment. Identical residues are shown in white on a red background while similar residues are shown in red. Sequences were plotted with ESPript 2.2 [41]. B: Modeling of R388 StbB 3D structure. Left: Crystal structure of Soj (top) and model of StbB (bottom) dimers bound to ATP. Yellow and blue ribbons represent the two monomers. Right: close-up of the P-loop in the Soj structure showing the active site which accommodates two molecules of ATP. The lysine 15 (K15) which stabilizes the negative charges on the opposing ATP [16] is indicated. In the StbB model, the polar residue serine 9 (S9) could play the same role as Soj K15. StbB structure was modeled by QuickPhyre [42] and Pymol DeLano, W.L. PyMOL Molecular Graphics System (2002) DeLano Scientific, San Carlos, CA, USA (http://www.pymol.org) was used to prepare the figure.
(PDF)

Figure S2 Sequence alignment of StbA and homologous proteins. The top sequence and the numbering represent R388 StbA. Predicted alpha-helices are shown above the alignment. Identical residues are shown in white on a red background while similar residues are shown in red. Sequences were plotted with ESPript 2.2 [41].
(PDF)

Figure S3 Protein StbA specifically binds DNA fragments containing *stbDRs in vitro*. Electrophoretic shift assay (EMSA) of *stbDRs* with StbA-His_6_ protein. A 200 bp ^32^P-labelled PCR fragment containing R388 *stbDRs* was incubated at 30°C for 20 min with various amounts of StbA in the presence of non-specific DNA and the products separated by electrophoresis in a 4% polyacrylamide gel in TB buffer (Text S1). Unbound DNA and StbA-DNA complexes are shown by open and filled arrowheads respectively. Lane 1: StbA omitted; Lanes 2, 3, and 4: 250, 500, and 1000 nM of StbA, respectively.
(PDF)

Figure S4 Localization of plasmid R388 and derivatives in live *E. coli* cells relative to nucleoid position. LN2666 cells were prepared as described in Figure 6 and then chromosomal DNA was stained with DAPI (Materials and Methods). From left to right: overlay phase/GFP-ParB (green); chromosomal DNA (DAPI in blue); overlay phase/chromosomal DNA/GFP-ParB (blue/green). A: R388; B: R388ΔΔ*stbA*; C: R388Δ: R388Δ*stbB*.
(PDF)

Table S1 Bacterial strains and plasmids.
(PDF)

Table S2 Oligonucleotides used in this study.
(PDF)

Text S1 Supplementary experimental procedures.
(PDF)

## References

[1] de la Cruz F, Davies J (2000). Horizontal gene transfer and the origin of species: lessons from bacteria.. Trends Microbiol.

[2] Bouet JY, Nordstrom K, Lane D (2007). Plasmid partition and incompatibility—the focus shifts.. Mol Microbiol.

[3] Ebersbach G, Gerdes K (2005). Plasmid segregation mechanisms.. Annu Rev Genet.

[4] Salje J (2010). Plasmid segregation: how to survive as an extra piece of DNA.. Critical reviews in Biochemistry and Molecular Biology.

[5] Schumacher MA (2007). Structural biology of plasmid segregation proteins.. Curr Opin Struct Biol.

[6] de la Cruz F, Frost LS, Meyer RJ, Zechner EL (2010). Conjugative DNA metabolism in Gram-negative bacteria.. FEMS Microbiol Rev.

[7] Alvarez-Martinez CE, Christie PJ (2009). Biological diversity of prokaryotic type IV secretion systems.. Microbiol Mol Biol Rev.

[8] Cabezon E, de la Cruz F (2006). TrwB: an F(1)-ATPase-like molecular motor involved in DNA transport during bacterial conjugation.. Res Microbiol.

[9] Cascales E, Christie PJ (2004). Agrobacterium VirB10, an ATP energy sensor required for type IV secretion.. Proc Natl Acad Sci U S A.

[10] Tait RC, Close TJ, Rodriguez RL, Kado CI (1982). Isolation of the origin of replication of the IncW-group plasmid pSa.. Gene.

[11] Tait RC, Lundquist RC, Kado CI (1982). Genetic map of the crown gall suppressive IncW plasmid pSa.. Mol Gen Genet.

[12] Fernandez-Lopez R, Garcillan-Barcia MP, Revilla C, Lazaro M, Vielva L (2006). Dynamics of the IncW genetic backbone imply general trends in conjugative plasmid evolution.. FEMS Microbiol Rev.

[13] Koonin EV (1993). A common set of conserved motifs in a vast variety of putative nucleic acid-dependent ATPases including MCM proteins involved in the initiation of eukaryotic DNA replication.. Nucleic Acids Res.

[14] Gerdes K, Howard M, Szardenings F (2010). Pushing and pulling in prokaryotic DNA segregation.. Cell.

[15] Shih YL, Rothfield L (2006). The bacterial cytoskeleton.. Microbiol Mol Biol Rev.

[16] Leonard TA, Butler PJ, Lowe J (2005). Bacterial chromosome segregation: structure and DNA binding of the Soj dimer—a conserved biological switch.. Embo J.

[17] Datsenko KA, Wanner BL (2000). One-step inactivation of chromosomal genes in Escherichia coli K-12 using PCR products.. Proc Natl Acad Sci U S A.

[18] Bolland S, Llosa M, Avila P, de la Cruz F (1990). General organization of the conjugal transfer genes of the IncW plasmid R388 and interactions between R388 and IncN and IncP plasmids.. J Bacteriol.

[19] Paterson ES, More MI, Pillay G, Cellini C, Woodgate R (1999). Genetic analysis of the mobilization and leading regions of the IncN plasmids pKM101 and pCU1.. J Bacteriol.

[20] Ziegelin G, Pansegrau W, Lurz R, Lanka E (1992). TraK protein of conjugative plasmid RP4 forms a specialized nucleoprotein complex with the transfer origin.. J Biol Chem.

[21] Li Y, Austin S (2002). The P1 plasmid is segregated to daughter cells by a 'capture and ejection' mechanism coordinated with Escherichia coli cell division.. Mol Microbiol.

[22] Nielsen HJ, Li Y, Youngren B, Hansen FG, Austin S (2006). Progressive segregation of the Escherichia coli chromosome.. Mol Microbiol.

[23] Gordon GS, Sitnikov D, Webb CD, Teleman A, Straight A (1997). Chromosome and low copy plasmid segregation in E. coli: visual evidence for distinct mechanisms.. Cell.

[24] Gordon S, Rech J, Lane D, Wright A (2004). Kinetics of plasmid segregation in Escherichia coli.. Mol Microbiol.

[25] Niki H, Hiraga S (1997). Subcellular distribution of actively partitioning F plasmid during the cell division cycle in E. coli.. Cell.

[26] Pogliano J, Ho TQ, Zhong Z, Helinski DR (2001). Multicopy plasmids are clustered and localized in Escherichia coli.. Proc Natl Acad Sci U S A.

[27] Sengupta M, Nielsen HJ, Youngren B, Austin S (2010). P1 plasmid segregation: accurate redistribution by dynamic plasmid pairing and separation.. J Bacteriol.

[28] Judd PK, Kumar RB, Das A (2005). Spatial location and requirements for the assembly of the Agrobacterium tumefaciens type IV secretion apparatus.. Proc Natl Acad Sci U S A.

[29] Judd PK, Kumar RB, Das A (2005). The type IV secretion apparatus protein VirB6 of Agrobacterium tumefaciens localizes to a cell pole.. Mol Microbiol.

[30] Kumar RB, Das A (2002). Polar location and functional domains of the Agrobacterium tumefaciens DNA transfer protein VirD4.. Mol Microbiol.

[31] Teng WL, Bannam TL, Parsons JA, Rood JI (2008). Functional characterization and localization of the TcpH conjugation protein from Clostridium perfringens.. J Bacteriol.

[32] Erdmann N, Petroff T, Funnell BE (1999). Intracellular localization of P1 ParB protein depends on ParA and parS.. Proc Natl Acad Sci U S A.

[33] Jensen RB, Gerdes K (1999). Mechanism of DNA segregation in prokaryotes: ParM partitioning protein of plasmid R1 co-localizes with its replicon during the cell cycle.. Embo J.

[34] Lawley TD, Taylor DE (2003). Characterization of the double-partitioning modules of R27: correlating plasmid stability with plasmid localization.. J Bacteriol.

[35] Atmakuri K, Cascales E, Burton OT, Banta LM, Christie PJ (2007). Agrobacterium ParA/MinD-like VirC1 spatially coordinates early conjugative DNA transfer reactions.. Embo J.

[36] Garner EC, Campbell CS, Weibel DB, Mullins RD (2007). Reconstitution of DNA segregation driven by assembly of a prokaryotic actin homolog.. Science.

[37] Ringgaard S, van Zon J, Howard M, Gerdes K (2009). Movement and equipositioning of plasmids by ParA filament disassembly.. Proc Natl Acad Sci U S A.

[38] Cornet F, Mortier I, Patte J, Louarn JM (1994). Plasmid pSC101 harbors a recombination site, psi, which is able to resolve plasmid multimers and to substitute for the analogous chromosomal Escherichia coli site dif.. J Bacteriol.

[39] Khlebnikov A, Datsenko KA, Skaug T, Wanner BL, Keasling JD (2001). Homogeneous expression of the P(BAD) promoter in Escherichia coli by constitutive expression of the low-affinity high-capacity AraE transporter.. Microbiology.

[40] Yates P, Lane D, Biek DP (1999). The F plasmid centromere, sopC, is required for full repression of the sopAB operon.. J Mol Biol.

[41] Gouet P, Courcelle E, Stuart DI, Metoz F (1999). ESPript: analysis of multiple sequence alignments in PostScript.. Bioinformatics.

[42] Kelley LA, Sternberg MJ (2009). Protein structure prediction on the Web: a case study using the Phyre server.. Nat Protoc.

