# Supplementary figures and images for: The stb Operon Balances the Requirements for Vegetative Stability and Conjugative Transfer of Plasmid R388
Source: PLoS Genet. 2011 May 19;7(5):e1002073. doi: 10.1371/journal.pgen.1002073 (PMC3098194; doi:10.1371/journal.pgen.1002073)

**A**

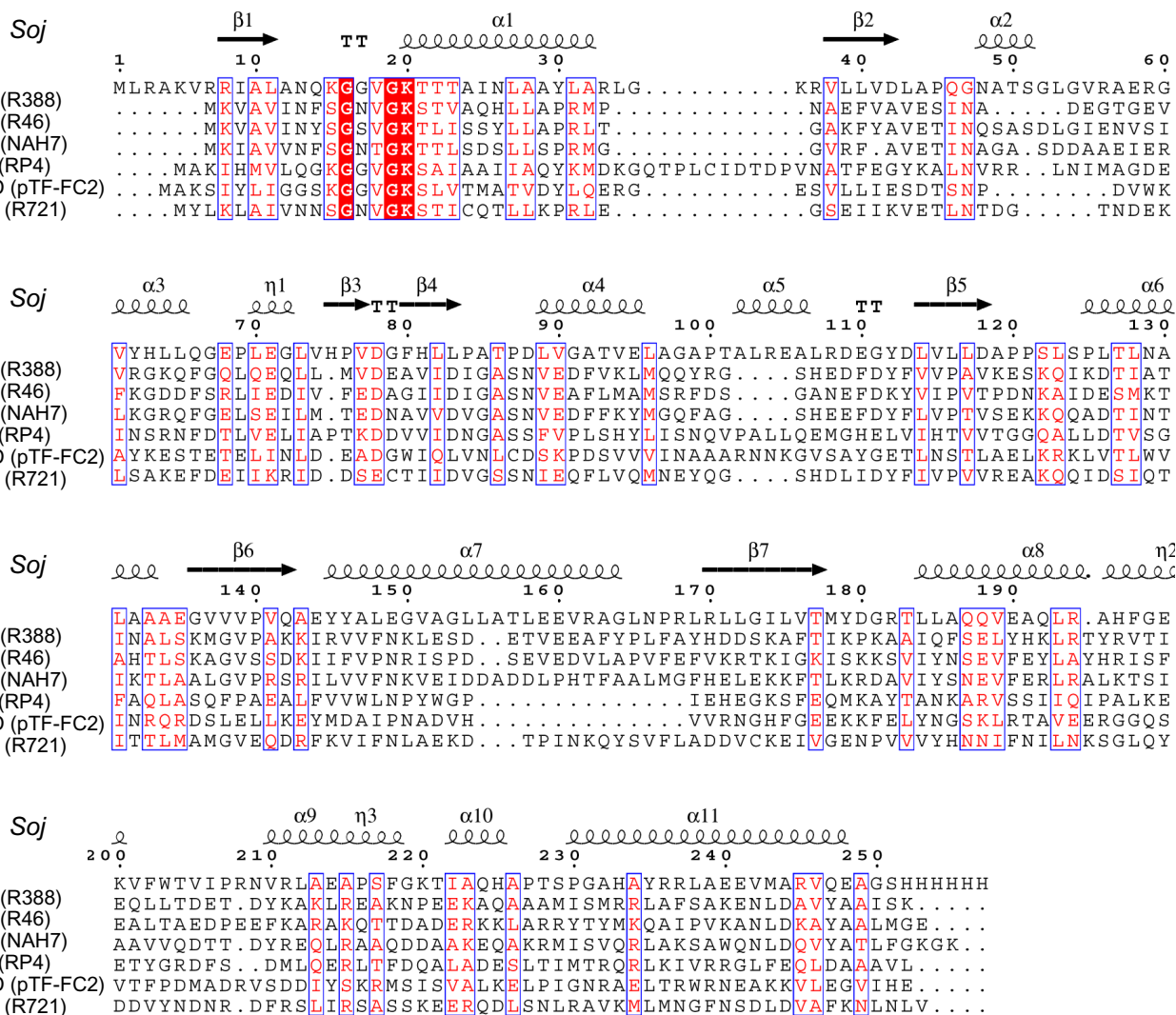

**B**

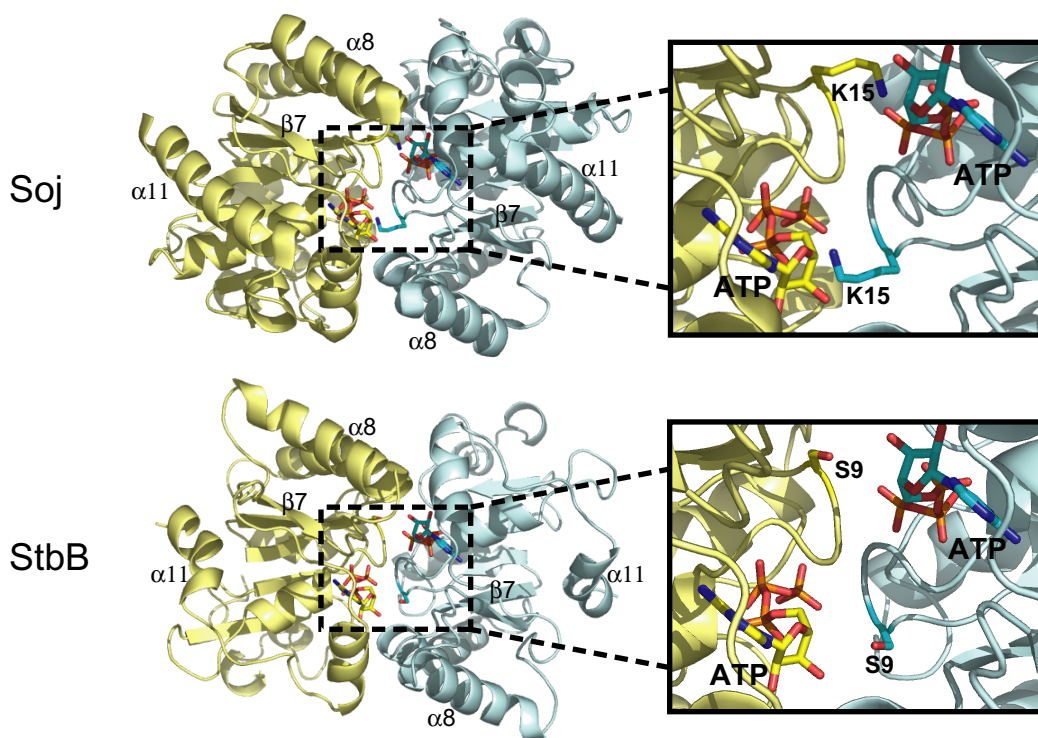

Supplement: Figure S1 — StbB proteins contain a putative deviant Walker A ATP-binding motif (P-loop). A: Sequence alignment of R388 StbB protein and homologs from representative plasmid families. The sequence and the numbering highlighted above the alignment are from the crystal structure of Soj (PDB ID: 2BEK). The secondary structure elements of Soj are also shown above the alignment. Identical residues are shown in white on a red background while similar residues are shown in red. Sequences were plotted with ESPript 2.2 [41]. B: Modeling of R388 StbB 3D structure. Left: Crystal structure of Soj (top) and model of StbB (bottom) dimers bound to ATP. Yellow and blue ribbons represent the two monomers. Right: close-up of the P-loop in the Soj structure showing the active site which accommodates two molecules of ATP. The lysine 15 (K15) which stabilizes the negative charges on the opposing ATP [16] is indicated. In the StbB model, the polar residue serine 9 (S9) could play the same role as Soj K15. StbB structure was modeled by QuickPhyre [42] and Pymol DeLano, W.L. PyMOL Molecular Graphics System (2002) DeLano Scientific, San Carlos, CA, USA (http://www.pymol.org) was used to prepare the figure. (PDF) [file pgen.1002073.s001.pdf]

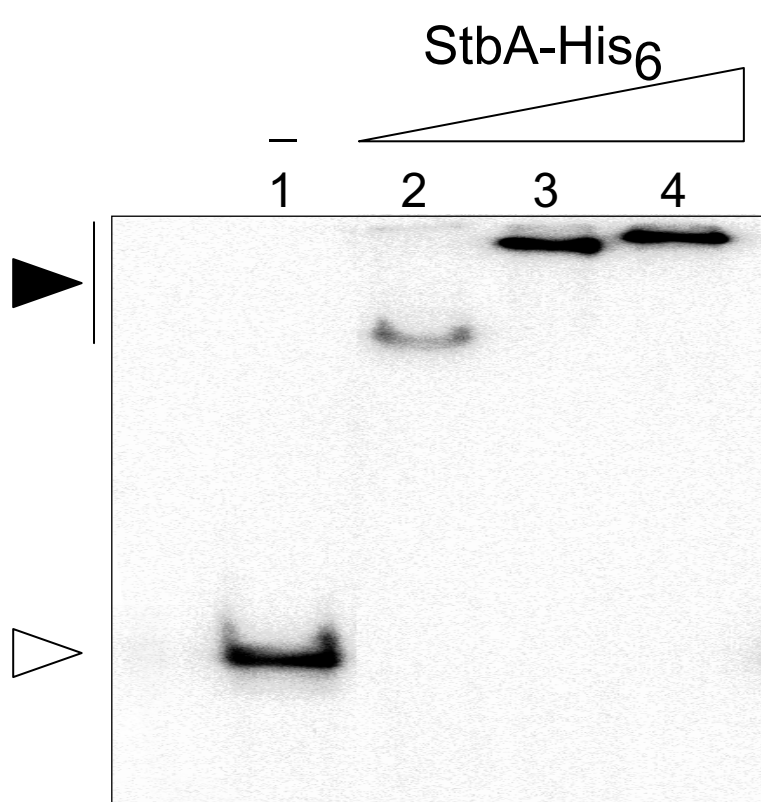

Supplement: Figure S3 — Protein StbA specifically binds DNA fragments containing stbDRs in vitro. Electrophoretic shift assay (EMSA) of stbDRs with StbA-His6 protein. A 200 bp 32P-labelled PCR fragment containing R388 stbDRs was incubated at 30°C for 20 min with various amounts of StbA in the presence of non-specific DNA and the products separated by electrophoresis in a 4% polyacrylamide gel in TB buffer (Text S1). Unbound DNA and StbA-DNA complexes are shown by open and filled arrowheads respectively. Lane 1: StbA omitted; Lanes 2, 3, and 4: 250, 500, and 1000 nM of StbA, respectively. (PDF) [file pgen.1002073.s003.pdf]

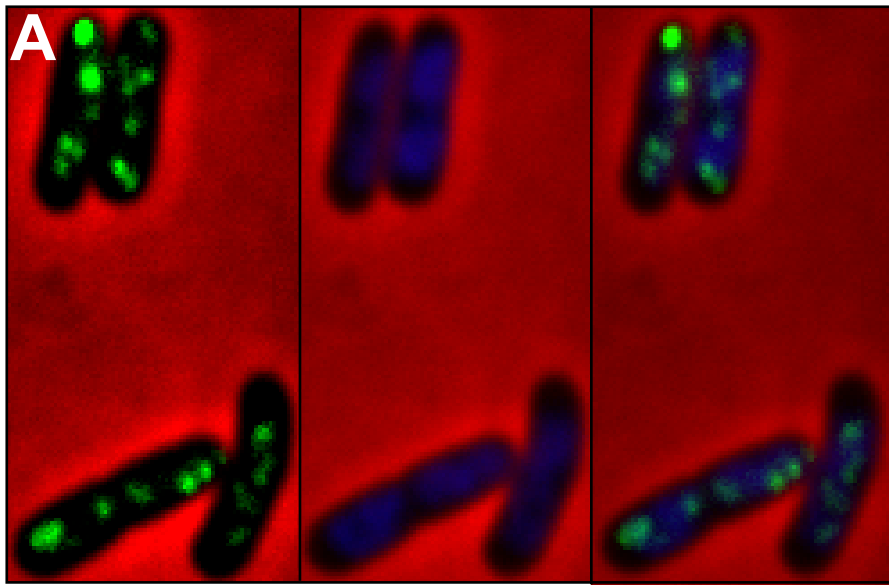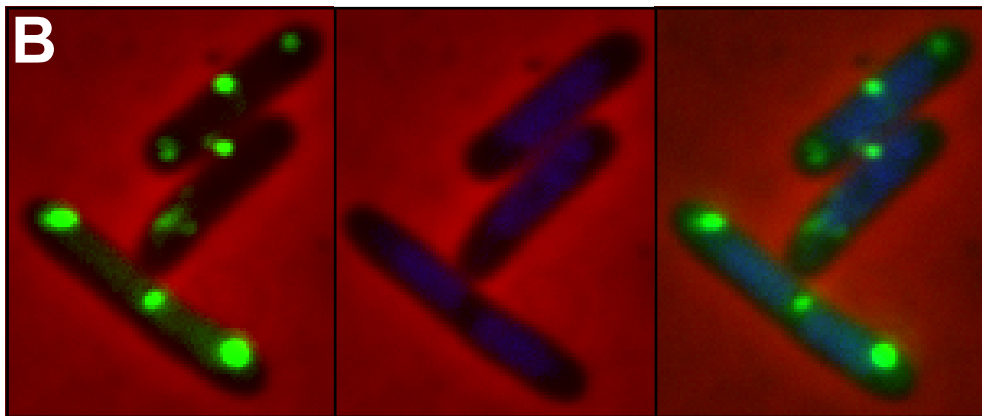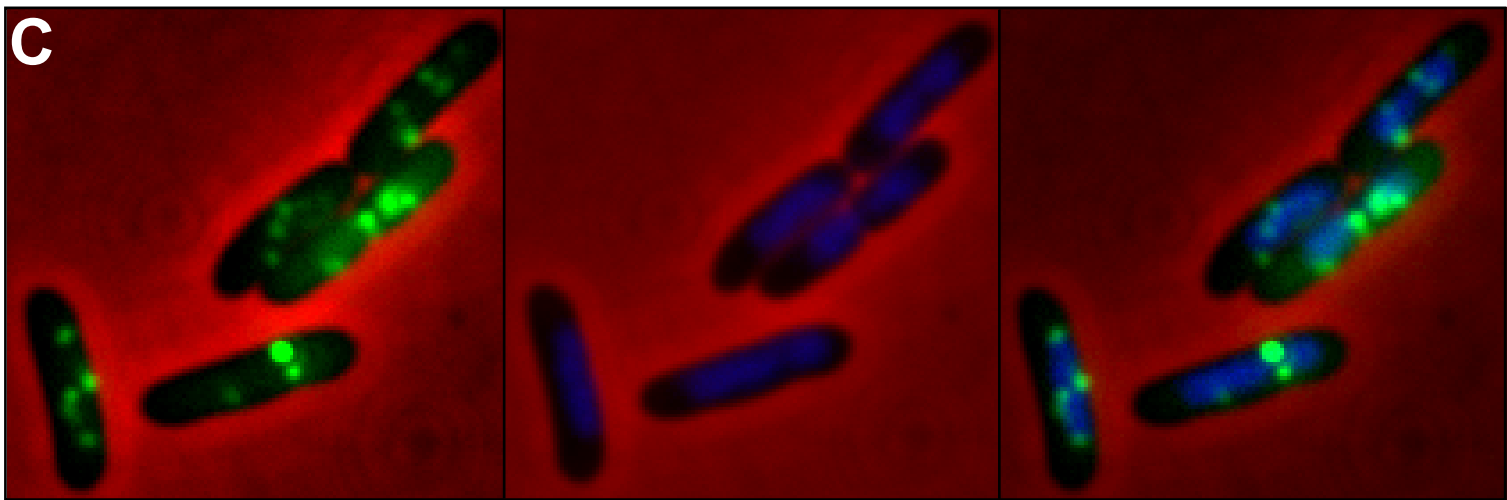

Supplement: Figure S4 — Localization of plasmid R388 and derivatives in live E. coli cells relative to nucleoid position. LN2666 cells were prepared as described in Figure 6 and then chromosomal DNA was stained with DAPI (Materials and Methods). From left to right: overlay phase/GFP-ParB (green); chromosomal DNA (DAPI in blue); overlay phase/chromosomal DNA/GFP-ParB (blue/green). A: R388; B: R388ΔΔstbA; C: R388Δ: R388ΔstbB. (PDF) [file pgen.1002073.s004.pdf]
