# Supplementary material for: The stb Operon Balances the Requirements for Vegetative Stability and Conjugative Transfer of Plasmid R388
Source: PLoS Genet. 2011 May 19;7(5):e1002073. doi: 10.1371/journal.pgen.1002073 (PMC3098194; doi:10.1371/journal.pgen.1002073)
Supplement: Table S1 — Bacterial strains and plasmids. (PDF) [file pgen.1002073.s005.pdf]

**Strains**	**Genotype/relevant properties**	**AB- res**	**Source/reference**
DH5α	F^- *end*A1 *rec*A1 *gyr*A96 *thi*-1 *hsd*R17 *sup*E44 *rel*A1 Δ(*arg*F- *lac*ZYA) U169 Φ80*d lac*Z D*M15 gyr*A96	Nx	Sambrook et al., 1989
LN2666	F^- W1485 *thi*A *thy*A *leu deo*B *rps*L	Sm	[38]
BW27783	BW25113 DE(*ara*FGH) F(Δ*araEp* P_{CP8}-*ara*E)	Rif	[39]
DY380	DH10B derivative containing a defective λ prophage; *red*, *bet* and *gam* genes are controlled by the temperature-sensitive λcI857 repressor	Sm	Lee et al., 2001
C41 (DE3)	F^- *omp*T *dcm hsd*S (r_{B-} m_{B-}) *galλ* (DE3)		Miroux and Walker, 1996
**Plasmids**			
R388	R388 *parS1-Cm*	Tmp,Cm	This study
R388*parS2-Cm*	R388*parS2-Cm*	Tmp,Cm	This study
R388Δ*stbA*	R388 *parS-Cm* Δ(*stbA*)	Tmp,Cm	This study
R388Δ*stbB*	R388 *parS-Cm* Δ(*stbB*)	Tmp,Cm	This study
R388Δ*stbC*	R388 *parS-Cm* Δ(*stbC*)	Tmp,Cm	This study
R388Δ*stbABC*	R388 *parS-Cm* Δ(*stbABC*)	Tmp,Cm	This study
R388Δ(*it-stbABC*)	R388 *parS-Cm* Δ(*it-stbABC*)	Tmp,Cm	This study
R388Δ(*it-kfrA*)	R388 *parS-Cm* Δ(*it-kfrA*)	Tmp,Cm	This study
pAPT110	Used to amplify the Km resistance gene (p15A), empty vector (V) for conjugation and plasmid stability assays)	Km, Sp/Sm	Polard and Chanlder, 1995
pStbA	pAPT110 derivative expressing *stbA* from P*lac* promoter	Sp/Sm	This study
pStbB	pAPT110 derivative expressing *stbB* from P*lac* promoter	Sp/Sm	This study
pET-StbA	pET29C (Novagen) derivative expressing *stbA*	Km	This study
pALA2705	*GFP-ΔN30parB* expressing from P*lac* promoter	Ap	[21]
pGBKD3-*parS*	contains the *parS* sequence from the P1 bacteriophage adjacent to the Cm resistance gene	Cm	Espeli et al.,2008

Table S1. Bacterial strains and plasmids.
