# Supplementary material for: The stb Operon Balances the Requirements for Vegetative Stability and Conjugative Transfer of Plasmid R388
Source: PLoS Genet. 2011 May 19;7(5):e1002073. doi: 10.1371/journal.pgen.1002073 (PMC3098194; doi:10.1371/journal.pgen.1002073)
Supplement: Table S2 — Oligonucleotides used in this study. (PDF) [file pgen.1002073.s006.pdf]

Name	Sequence 5'→ 3'
G3	AGGTACCATATGAATGAAACGGACGC
G16	TCCTGTTAGTTAAAGGTCGTTTGC
G17	CTCCCAATGCGCTATGCGCATTGG
G18	AGGCAAACGACCTTTAACTAACAGGAGGGCTACAAGTGAATGAAACGGACGGT ACCCATCATGAACAATAAAACTGTCTGC
G19	CCCGAAAAATTGATCACAGCTACTTTCATTTGTTGTCACTCCGGGTACCCAGTGT TACAACCAATTAACC
G20	CCTTAAACGGCCTATTTTTTCCAAGCGGAGTGACAACAAATGAAAGTAGCTGTG GGTACCCATCATGAACAATAAAACTGTCTGC
G21	GCGGGCCAGCCATAAGCTATCCCCCGTTACTTGGAGATAGCGGCGGTACCCAGT GTTACAACCAATTAACC
G22	CGATGCGGTCTATGCCGCTATCTCCAAGTAACGGGGGATAGCTTATGGCTGGCCC GCAAGGTACCCATCATGAACAATAAAACTGTCTGC
G23	GGAGGGTGAGGGGGGAGAGGGGCGGCCCCGCAGGGCCGCCAGTTCAAGCGCCG AAGAAGTAGGTACCCAGTGTTACAACCAATTAACC
G25	AGGTACCATATGAAAGTAGCTGTGATCAA
G44	CAGCGTGACCCTAAAGAGGGTCAAAACTGCTCCCAATGCGCTATGCGCATGGTA CCCATCATGAACAATAAAACTGTCTGC
G49	GCAAGTATGATGCAAACGGGGCTTTATCGTCAATAGCTGTCAATAGATCGCTGT GTAGGCTGGAGCTGCTTCG
G50	CCATCTACCAAGAAGGAAACGGGCAAATAGCATTCGGATTTTGAAACGCTGGTC TGCTATGTGGTGCTATCT
G68	GTATGTAGTATCATACAACATACTACAGTACAGAGGCCCGCAAGAATGGCAATC ACTAAAGGTACCCAGTGTTACAACCAATTAACC
G73	TGATCCAGCATGCGTCGACTTACTTGGAGATAGCGGCATAG
G78	ATGCGGATCCTCACTCACTCGCTCGCGCTTTCTTGC
G79	GGGCTTTTCGGGCCTGTCAGGCTTGCTCGGCCGCCTCTGCGACTTCGCTGTGTAG GCTGGAGCTGCTTCG
dxsF	CGAGAAACTGGCGATCCTTA
dxsR	CTTCATCAAGCGGTTTCACA
resPleft	CGAGTTGTTAGGCGTCAGC
resPright	GCCTTCGTTTAGGTGCTGAA
StbAN	AAAAACATATGGTGAATGAAACG
StbAX	AAAAACTCGAGTTTGTTGTCACT

Table S2. Oligonucleotides used in this study.
