# Supplementary material for: The stb Operon Balances the Requirements for Vegetative Stability and Conjugative Transfer of Plasmid R388
Source: PLoS Genet. 2011 May 19;7(5):e1002073. doi: 10.1371/journal.pgen.1002073 (PMC3098194; doi:10.1371/journal.pgen.1002073)
Supplement: Figure S2 — Sequence alignment of StbA and homologous proteins. The top sequence and the numbering represent R388 StbA. Predicted alpha-helices are shown above the alignment. Identical residues are shown in white on a red background while similar residues are shown in red. Sequences were plotted with ESPript 2.2 [41]. (PDF) [file pgen.1002073.s002.pdf]

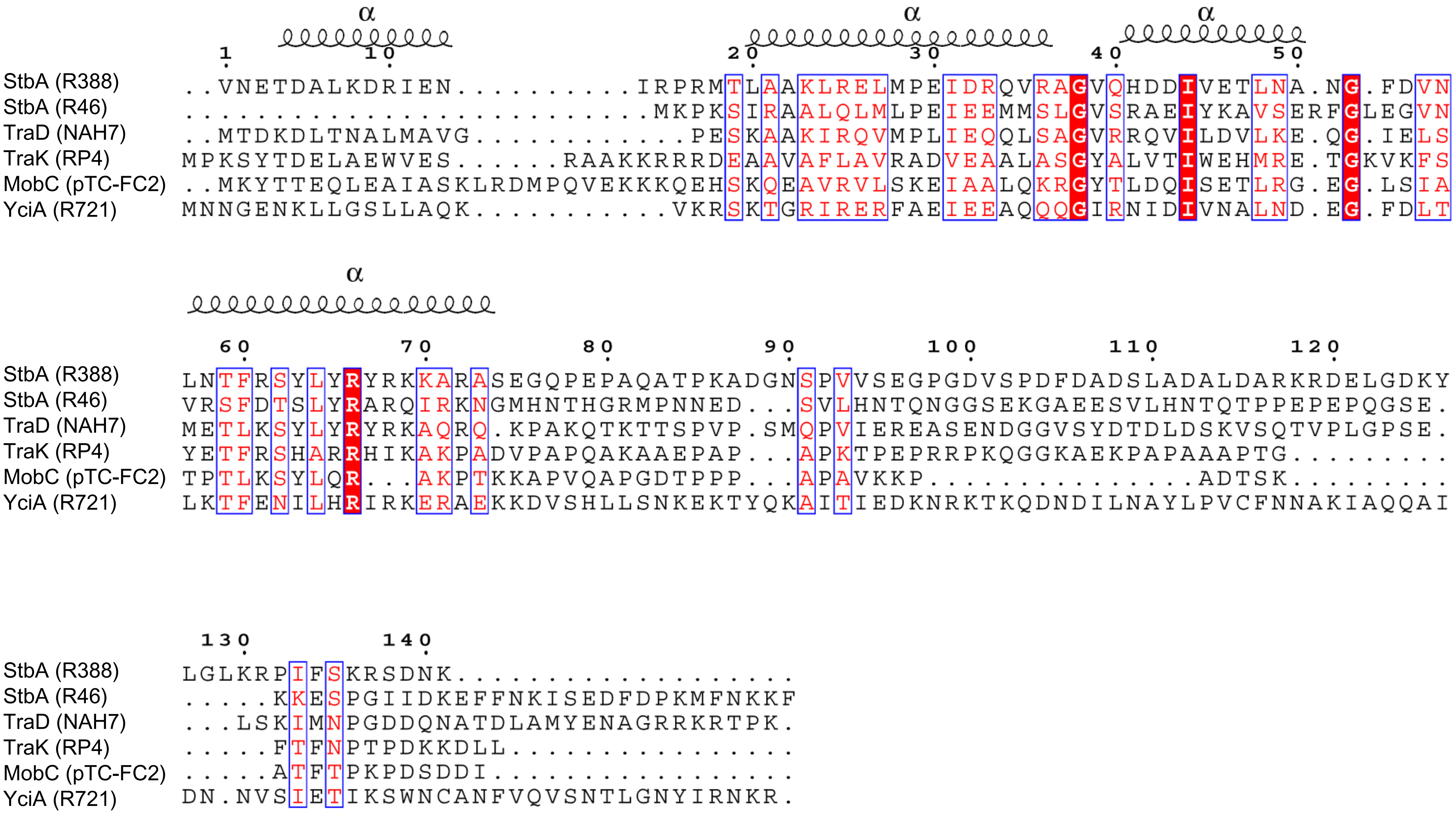
α
α
α
1 10 20 30 40 50
StbA (R388) ..VNETDALKDRIEN..........IRPRMTLAAKLRELMPEIDRQVRAGVQHDDIVETLNA.NG.FDVN
StbA (R46) ..........................MKPKSIRAALQLMLPEIEEMMSLGVSRAEIYKAVSERFGLEGVN
TraD (NAH7) ..MTDKDLTNALMAVG............PESKAAKIRQVMPLIEQQLSAGVRRQVILDVLKE.QG.IELS
TraK (RP4) MPKSYTDELAEWVES......RAAKKRRRDEAAVAFLAVRADVEAALASGYALVTIWEHMRE.TGKVKFS
MobC (pTC-FC2) ..MKYTTEQLEAIASKLRDMPQVEKKKQEHSKQEAVRVLSKEIAALQKRGYTLDQISETLRG.EG.LSIA
YciA (R721) MNNGENKLLGSLLAQK...........VKRSKTGRIRERFAEIEEAQQQGIRNIDIVNALND.EG.FDLT
α
60 70 80 90 100 110 120
StbA (R388) LNTFRSYLYRYRKKARASEGQPEPAQATPKADGNSPVVSEGPGDVSPDFDADSLADALDARKRDELGDKY
StbA (R46) VRSFDTSLYRARQIRKNGMHNTHGRMPNNED...SVLHNTQNGGSEKGAEESVLHNTQTPPEPEPQGSE.
TraD (NAH7) METLKSYLYRYRKAQRQ.KPAKQTKTTSPVP.SMQPVIEREASENDGGVSYDTDLDSKVSQTVPLGPSE.
TraK (RP4) YETFRSHARRHIKAKPADVPAPQAKAAEPAP...APKTPEPRRPKQGGKAEKPAPAAAPTG.........
MobC (pTC-FC2) TPTLKSYLQR...AKPTKKAPVQAPGDTPPP...APAVKKP...............ADTSK.........
YciA (R721) LKTFENILHRIRKERAEKKDVSHLLSNKEKTYQKAITIEDKNRKTKQDNDILNAYLPVCFNNAKIAQQAI
130 140
StbA (R388) LGLKRPIFSKRSDNK...................
StbA (R46) .....KKESPGIIDKEFFNKISEDFDPKMFNKKF
TraD (NAH7) ...LSKIMNPGDDQNATDLAMYENAGRRKRTPK.
TraK (RP4) .....FTFNPTPDKKDLL................
MobC (pTC-FC2) .....ATFTPKPDSDDI.................
YciA (R721) DN.NVSIETIKSWNCANFVQVSNTLGNYIRNKR.
