# Supplementary material for: The stb Operon Balances the Requirements for Vegetative Stability and Conjugative Transfer of Plasmid R388
Source: PLoS Genet. 2011 May 19;7(5):e1002073. doi: 10.1371/journal.pgen.1002073 (PMC3098194; doi:10.1371/journal.pgen.1002073)
Supplement: Text S1 — Supplementary experimental procedures. (PDF) [file pgen.1002073.s007.pdf]

Text S1. Supplementary experimental procedures

Bacterial strains and plasmids

Bacterial strains and plasmids used in this study are listed in table S1, and oligonucleotides in table S2. *stbA* and *stbB* genes were amplified by PCR from plasmid R388 with oligonucleotides G3 and G72, and G25 and G73, respectively, restricted with *Nde*I and *Sph*I and inserted into a pAPT110 derivative plasmid (Ton-Hoang et al. 2005). The *stbA* gene was amplified by PCR from plasmid R388 using oligonucleotides StbAN and StbAX, and placed between *Nde*I and *Xho*I sites of plasmid pET29C (Novagen). The resulting recombinant protein is a histidine-tagged/StbA fusion. R388 derivatives were constructed using phage λ red-mediated gene recombination using DY380 strain (Yu et al. 2000). DNA substrates were generated through PCR amplification with primers that produced a linear resistance cassette surrounded by two *Kpn*I restriction sites and at least 50 bp terminal arms homologous to the desired target site. R388*parS* and R388*parS2* were constructed by insertion of a *parS-Cm* resistance cassette, amplified from pGBKD3-*parS* (Espeli et al. 2008) which contains the *parS* sequence from the P1 bacteriophage adjacent to the Cm resistance gene, into intergenic regions of R388 (R388*parS*: between *orf5* and *korB*; R388*parS2*: between *orf14* and *klcB*). R388 deletions were constructed by replacement of the corresponding gene with a Km resistance cassette amplified from pAPT110 (Polard and Chandler 1995). DNA substrates were introduced by electroporation into DY380 strain harbouring R388 were grown as described in Yu et al. (Yu et al. 2000; Lee et al. 2001). Cells were plated on agar plates containing the appropriate antibiotic to select for the desired insertions. To screen the presumptive colonies, the target gene was PCR amplified and analyzed by 1% agarose electrophoresis. For R388 deletions, the Km cassettes were removed by

digestion with *Kpn*I followed by self-ligation of the resulting plasmid. Constructions were all verified by sequencing of a PCR product of the corresponding region. Primers used were as follows: to create DNA templates to insert the *parS* site, G49 and G50 (R388*parS*) and G78 and G79 (R388*parS2*); to create DNA templates to delete *stbABC*, G18 and G23; to create DNA templates to delete (*stbDRs*-*stbABC*), G44 and G23; to create DNA templates to delete *stbA*, G18 and G19; to create DNA templates to delete *stbB*, G20 and G21; to create DNA templates to delete *stbC*, G22 and G23; to create DNA templates to delete (*stbDRs-kfrA*), G44 and G68; to create DNA templates to delete *stbABC-kfrA*, G18 and G68.

General procedures

Luria–Bertani (LB) broth was used for bacterial growth (Sambrook 1989). For microscopy, M9 medium supplemented with 0.2% casamino acids, 0.4% glucose, 2 µg/ml thiamine, 20 µg/ml leucine and 20 µg/ml thymine was used. Selective media included antibiotics as needed at the following concentrations: ampicillin(Ap), 100 µg/ml; chloramphenicol (Cm), 25 µg/ml; Kanamycin (Km), 25 µg/ml; nalidixic acid (Nx), 20 µg/ml; streptomycin (Sm), 300 µg/ml; spectinomycin (Sp), 100 µg/ml; trimethoprim (Tp), 20 µg/ml. Plasmid DNA was extracted using Sigma GenElute Miniprep kit. PCR products were purified using QIAquick Gel Extraction Kit (Quiagen). Bacterial transformation with plasmid DNA was carried out by electroporation. Strain DH5α (Sambrook 1989) was used for general plasmid construction and maintenance; strains LN2666 (Cornet et al. 1994) and BW27783 (Khlebnikov et al. 2001) were used for conjugation experiments. Restriction endonucleases, Vent DNA polymerase and T4 DNA ligase were from Fermentas or New England Biolabs. Primer oligonucleotides were purshased from Sigma.

Copy number determination by real-time quantitative PCR

The relative quantification method was used to quantify plasmid copy numbers as described in Lee et al. (Lee et al. 2006), using the *E. coli* chromosome single copy 1-deoxyxylulose-5-phosphate synthase gene (*dxs*) and plasmid R388 *resP* gene as the reference and target genes, respectively. *E. coli* strains LN2666 carrying the relevant plasmid were grown at 37ºC in 10 ml LB supplemented with appropriate antibiotics. The cells were harvested and total DNA was isolated using Instagene™ Matrix (BIO-RAD). 20 ng, 10 ng and 5 ng of the DNA preparation were used as templates for real-time qPCR for each reaction. Primer sets for the *dxs* gene (dxsF and dxsR) and the R388 gene (resPleft and resPright) were used to amplify the reference *dxs* and the R388 *resP* genes. Real-time qPCR amplification and analysis were performed using the iCycler iQ Multicolor Real-Time Detection System with software version 3.1 (Bio-Rad). The real-time qPCR mixture was prepared using iQ SYBR green Supermix (Bio-Rad). The protocol was: 3 min at 95ºC, 50 cycles of 10s at 95ºC, and 30 s at 57ºC.

StbA purification and electrophoretic mobility shift assay

R388 StbA was expressed in *E. coli* C41 (DE3) (Miroux and Walker 1996) using the pET29c vector (Novagen) and purified to homogeneity by HisTrap HP (GE healthcare) affinity chromatography and Superdex S75 (GE healthcare) gel-filtration chromatography. A DNA fragment of 200 bp containing the *stb*DRs was amplified by PCR from plasmid R388 with oligonulceotides G16 and G17, and 5'-end labelled with ^{32}P using T4 polynucleotide kinase (Biolabs). In a standard gel retardation assay, 1 nM of the DNA fragment was incubated with increasing concentrations of StbA-His_6 in a final volume of 10 µl of binding mixture (20mM Tris-HCl (pH7.5), 200mM NaCl, 1mM

DTT, 15% glycerol) with competitor DNA (2 μg denatured sperm salmon) for 20 min at 30ºC. Reaction mixtures were loaded onto a 4% non-denaturing polyacrylamide gel.

Supplemental references

Cornet F, Mortier I, Patte J, Louarn JM (1994) Plasmid pSC101 harbors a recombination site, psi, which is able to resolve plasmid multimers and to substitute for the analogous chromosomal Escherichia coli site dif. Journal of bacteriology 176(11): 3188-3195.

Espeli O, Mercier R, Boccard F (2008) DNA dynamics vary according to macrodomain topography in the E. coli chromosome. Molecular microbiology 68(6): 1418-1427.

Khlebnikov A, Datsenko KA, Skaug T, Wanner BL, Keasling JD (2001) Homogeneous expression of the P(BAD) promoter in Escherichia coli by constitutive expression of the low-affinity high-capacity AraE transporter. Microbiology (Reading, England) 147(Pt 12): 3241-3247.

Lee CL, Ow DS, Oh SK (2006) Quantitative real-time polymerase chain reaction for determination of plasmid copy number in bacteria. Journal of microbiological methods 65(2): 258-267.

Lee EC, Yu D, Martinez de Velasco J, Tessarollo L, Swing DA et al. (2001) A highly efficient Escherichia coli-based chromosome engineering system adapted for recombinogenic targeting and subcloning of BAC DNA. Genomics 73(1): 56-65.

Miroux B, Walker JE (1996) Over-production of proteins in Escherichia coli: mutant hosts that allow synthesis of some membrane proteins and globular proteins at high levels. Journal of molecular biology 260(3): 289-298.

Polard P, Chandler M (1995) An in vivo transposase-catalyzed single-stranded DNA circularization reaction. Genes & development 9(22): 2846-2858.

Sambrook J, Fritsch, E.F., and Maniatis, T. (1989) Molecular Cloning: A Laboratory Manual.

Ton-Hoang B, Guynet C, Ronning DR, Cointin-Marty B, Dyda F et al. (2005) Transposition of ISHp608, member of an unusual family of bacterial insertion sequences. The EMBO journal 24(18): 3325-3338.

Yu D, Ellis HM, Lee EC, Jenkins NA, Copeland NG et al. (2000) An efficient recombination system for chromosome engineering in Escherichia coli. Proceedings of the National Academy of Sciences of the United States of America 97(11): 5978-5983.
